# Integrative multi-omics and mendelian randomization reveal the critical role of pyroptosis in prognosis and therapy of lung squamous cell carcinomas

**DOI:** 10.3389/fcell.2026.1822831

**Published:** 2026-07-08

**Authors:** Taihao Wang, Wei Zhang, Zhihao He, Jichun Song, Baichuan Cao, Qin Li, Cuizhen Fan

**Affiliations:** 1 School of Biomedical Engineering, Capital Medical University, Beijing, China; 2 Department of Oncology, Beijing Friendship Hospital, Capital Medical University, Beijing, China; 3 Department of Oncology, Beijing Chao-Yang Hospital, Capital Medical University, Beijing, China

**Keywords:** lung squamous cell carcinomas, multi-omics, programmed cell death, pyroptosis, summary-data-based mendelian randomization

## Abstract

**Background:**

Most types of programmed cell death (PCD) have been demonstrated to play critical roles in the pathogenesis and prognosis of lung squamous cell carcinoma (LUSC). However, the specific type of PCD with the most prominent driving effect and regulatory value in LUSC remains unclear.

**Methods:**

Multi-omics data were integrated, and a multimodal autoencoder was employed to identify prognosis-related PCD gene modules and conduct weight ranking, leading to the construction of a PCD-associated neural network prognostic model. Summary-data-based Mendelian Randomization (SMR) analysis was applied to validate the causal relationship between key genes and LUSC. Combined with single-cell and spatial transcriptomics analyses, tumor–immune cell interactions were characterized, and the regulatory mechanism by which tumor cells modulate PCD in key immune cells through specific pathways was verified via cellular experiments.

**Results:**

Twelve prognosis-related PCD gene modules were identified in LUSC, among which the Pyroptosis_4 module emerged as the core risk signature. The prognostic model based on Pyroptosis_4 enabled effective risk stratification of early-stage patients. SMR analysis confirmed that the key pyroptosis gene NOD1 was directly associated with LUSC susceptibility, and NOD1 expression in M2 macrophages regulated the tumor immune microenvironment. Tumor cells formed a close spatial network with NOD1^-^ M2 macrophages and inhibited pyroptosis in M2 macrophages through the MDK/NCL signaling pathway.

**Conclusion:**

Pyroptosis plays a crucial role in the prognosis of LUSC, and this effect is associated with differential NOD1 expression in M2 macrophages. Tumor cells and NOD1^-^ M2 macrophages establish spatial interactions via the MDK/NCL pathway, emphasizing a potential candidate target for tumor immunoregulation-based strategies.

## Introduction

Lung cancer remains one of the leading causes of cancer-related death worldwide. Non-small cell lung cancer (NSCLC) accounts for approximately 85% of all lung cancer cases, while lung squamous cell carcinoma (LUSC) represents around 25%–30% of NSCLC cases (He et al., 2025). As a distinct histological subtype, LUSC is characterized by a relatively low driver gene mutation rate, severely limiting the availability of effective targeted therapies (Chen and Dhahbi, 2021). Although the combination of immune checkpoint inhibitors and chemotherapy has improved survival outcomes, patient responses remain highly heterogeneous, and only a small subset of patients achieve lasting responses to chemotherapy (Paz-Ares et al., 2020). Overall, treatment strategies for LUSC still lack sufficient precision and individualization. The dismal overall survival rate associated with this disease indicates that the identification of novel prognostic biomarkers and therapeutic targets remains a top research priority in this field.

Programmed cell death (PCD) involves a series of gene-regulated pathways that are essential for maintaining tissue homeostasis. It is an active and organized form of cell death determined by genes, including apoptosis, pyroptosis, ferroptosis, autophagy, and necroptosis. To date, approximately 18 well-documented PCD types have been identified, each defined by unique molecular mechanisms (Zhang et al., 2024a). Dysregulation of PCD enables cancer cells to evade death signals. Recent preclinical studies have highlighted the association between PCD and the development of LUSC. For instance, due to the lack of ferroptosis resistance mediated by glutathione peroxidase 4, LUSC exhibits increased sensitivity to artemisinin derivatives (Mölleken et al., 2025). Conversely, cancer cells can resist apoptosis through the expression of tripartite motif-containing 24 protein, thereby inhibiting ferroptosis (Sánchez-Ortega et al., 2025). Alterations in the PCD status of cancer cells can also affect the crosstalk with immune cells within the tumor microenvironment. Tumor cells undergoing necroptosis release damage-associated molecular patterns that activate CD8^+^ T cells and enhance their tumor-killing effects (Yatim et al., 2015). However, among the various forms of PCD, the specific types most closely associated with the prognosis of LUSC, as well as the therapeutic potential of cell-specific PCD, remain unclear.

**FIGURE 1:**
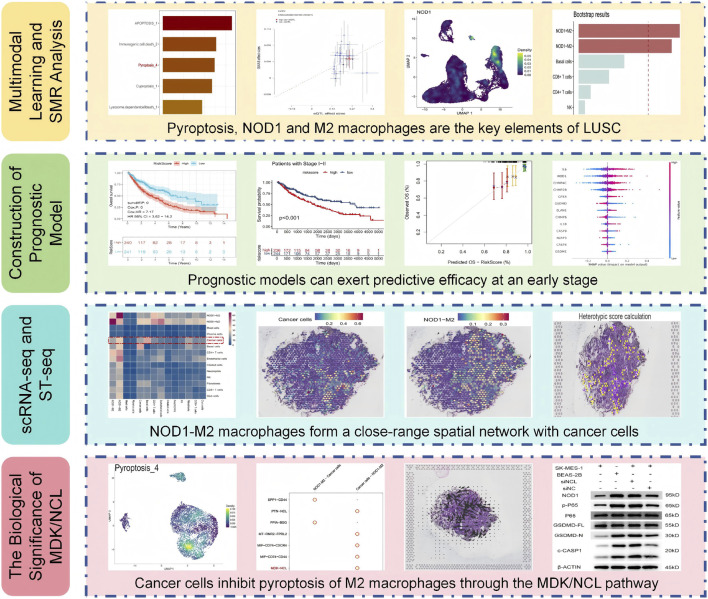
Workflow of data analysis and key conclusions of this study. This study integrated multi-omics data and identified pyroptosis as the core PCD type associated with LUSC prognosis. The constructed neural network model effectively stratified early-stage patients by risk. Combining Mendelian randomization and single-cell analysis, this study identified NOD1^-^ M2 macrophages as a key immune subset linked to LUSC initiation and progression, revealing increased infiltration rates in tumor tissues and further contributing to an immunosuppressive microenvironment. Spatial transcriptomics further revealed tight spatial interactions between NOD1^-^ M2 macrophages and cancer cells. Cellular experiments confirmed that tumor cells secrete MDK, which binds to the NCL receptor on M2 macrophages. This interaction inhibits NOD1-mediated pyroptosis, ultimately promoting tumor immune evasion. Therefore, the MDK/NCL pathway represents a potential candidate target.

In this study, genes associated with 18 forms of PCD were analyzed as distinct gene expression modalities, and prognosis-related multimodal features were screened using autoencoders and deep learning approaches. By integrating the results of summary-data-based Mendelian randomization (SMR) analysis, single-cell transcriptomics (scRNA-seq), and spatial transcriptomics (ST-seq), pyroptosis was identified as the core PCD type in LUSC, and a neural network prognostic model based on the PyTorch framework was subsequently constructed. Experimental studies further revealed that tumor cells educate M2 macrophages through intercellular communication, enhancing resistance to pyroptosis mediated by Nucleotide-Binding Oligomerization Domain Containing 1 (NOD1) and thereby maintaining an immunosuppressive tumor microenvironment (Figure 1). Overall, this study may help address the gap between basic PCD research and clinical translation, offering new therapeutic insights for patients with LUSC who have limited treatment options.

## Methods

### Data availability

Bulk RNA sequencing data used in this study were obtained from The Cancer Genome Atlas (TCGA) database (https://portal.gdc.cancer.gov/) and the Gene Expression Omnibus (GEO) database (https://www.ncbi.nlm.nih.gov/geo/). The TCGA-LUSC, GSE73403 and GSE74777 datasets were analyzed using a semi-supervised deep learning framework based on an autoencoder. After excluding samples with follow-up times <30 days, 657 LUSC samples and 51 normal lung tissue samples were retained. Aiming to eliminate non-technical batch effects across datasets, the “ComBat” algorithm implemented in the R package “sva” was applied. Differentially expressed genes were identified using the R package “limma,” with thresholds set at |log2 fold change (logFC)| > 1 and adjusted P-value (P.adj) < 0.05 (Leek et al., 2012; Ritchie et al., 2015). Overall survival (OS) was the primary survival endpoint of this study. Single-cell sequencing data were obtained from the GEO database. After quality verification, 22 LUSC samples from the GSE148071 dataset and seven normal lung tissue samples from the GSE128033 dataset were included (Wu et al., 2021; Morse et al., 2019). Cell viability was assessed based on gene expression counts and mitochondrial gene content, and low-quality or dead cells were excluded using the following criteria: (1) each feature was expressed in at least 3 cells, and each cell had at least 200 detected features; (2) total RNA counts per cell ≥1,000, with ≥200 and ≤10,000 detected genes per cell; (3) mitochondrial gene content was ≤20%, and ribosomal gene content was ≤40%. The “vst” method in the FindVariableFeatures function was used to identify the top 2000 highly variable genes. Following principal component analysis and cell clustering to identify cell subsets, marker validation and manual annotation were performed using the R package “SingleR” and the CellMarker 2.0 database (Aran et al., 2019; Hu et al., 2023). Spatial transcriptomics data (10× Visium platform) were obtained from the EMBL-EBI database (https://www.ebi.ac.uk/, accession number: E-MTAB-13530), encompassing two LUSC spatial transcriptomics datasets (De Zuani et al., 2024). Mitochondrial and ribosomal genes were removed using the Seurat package, and only genes expressed in more than 10 spots were retained. Low-quality spots were excluded using the criteria of >300 detected genes and >500 total reads. Data normalization was then performed using SCTransform, and spatial gene expression patterns were visualized using the SpatialFeaturePlot function in Seurat. Spots with SPRS scores greater than zero were designated as positive. Genome-wide association study (GWAS) data for LUSC were sourced from the OpenGWAS database (https://opengwas.io/), including the ieu-a-989 dataset (genome-wide genotyping arrays of 7,704 European LUSC cases and 54,763 controls) and the ebi-a-GCST004750 dataset (genome-wide genotyping arrays of 7,426 European LUSC cases and 55,627 controls). Additionally, PCD-related genes were collected from the published literature, covering 18 PCD types: apoptosis, pyroptosis, ferroptosis, autophagy, necroptosis, cuproptosis, parthanatos, entotic cell death, netotic cell death, lysosome-dependent cell death, alkaliptosis, oxeiptosis, NETosis, immunogenic cell death, anoikis, paraptosis, methuosis, and entosis (Zhang et al., 2024a). A total of 1964 PCD-related genes were identified (Supplementary Table S1).

### Screening of multimodal prognostic features and construction of prognostic model based on autoencoder and deep learning

Following established protocols reported in the literature, data preprocessing was first conducted, and all datasets were normalized to ensure comparability across multiple sources (Zhang et al., 2024b). Subsequently, autoencoders were used to transform each gene set into a low-dimensional representations. Considering the number of genes and research requirements, four autoencoders were trained for each gene set to obtain sufficient feature data. An attention mechanism was then integrated to enhance the capture of prognosis-related information, facilitating the selection of a minimal yet biologically meaningful feature set significantly associated with survival in TCGA-LUSC. Finally, a robust deep neural network-based prognostic prediction model was established, and samples from GSE73403 and GSE74777 were used as an independent validation set to assess model performance. Given that neural network prognostic prediction models are often regarded as “black-box” models, the Shapley Additive exPlanation (SHAP) was introduced in this study (Lundberg and Lee, 2017). SHAP provides local and global interpretations and is widely applicable for explaining various machine learning methods (Wang et al., 2021). Feature rankings based on the importance of the prediction model were derived by quantifying the contribution of each gene to the model’s prediction results. Kaplan–Meier curves, generated using the R packages “survival” and “survminer,” were used to evaluate differences in OS across different groups. Finally, calibration curves were constructed using the rms package to evaluate the predictive performance of the model.

### Analysis of scRNA-seq and GWAS data

Following the removal of confounding genes, such as ribosomal and mitochondrial protein genes, the “plot.scRNA.outlier” tool was used to visualize the distribution of specific genes in the scRNA-seq data. Cell–cell communication analysis was performed using Seurat v4.4.0, and the AddModuleScore function was applied to calculate the target gene set scores for each cell (Stuart et al., 2019). Pseudotime analysis of specified genes or gene sets was performed using the Vector algorithm. ScPagwas analysis was conducted using the R package scPagwas (Ma et al., 2023). Specifically, SNPs were mapped to adjacent genes and linked to corresponding pathways. For each pathway, a genetic association pathway activity score was calculated by multiplying regression coefficients with weighted pathway activity scores within the corresponding cells. The Seurat package was used to compute the trait-related score (TRS) for each cell. Significant TRS scores were defined as those exceeding the 95th percentile of a null distribution generated from 1,000 trait-label permutations. Bootstrap bias estimation was then used to verify the statistical significance of associations between each cell subset and LUSC (Hou et al., 2025). Finally, differential cell analysis between the two cohorts (TCGA-LUSC and GSE148071) was performed using the Bayesian framework implemented in the R package BayesPrism (Chu et al., 2022).

### RNA sequencing analysis

The immune status of tumor samples across different groups was evaluated using the R package “ESTIMATE,” which quantified these statuses based on ESTIMATEScore, ImmuneScore, StromalScore, and TumorPurity (Yoshihara et al., 2013). GO and KEGG enrichment analyses of key genes were conducted using the “ClusterProfiler” and “org.Hs.e.g.,.db” R packages, respectively (Yu et al., 2012). The Tumor Immune Dysfunction and Exclusion (TIDE) platform (http://tide.dfci.harvard.edu) was used to predict patient responses to immunotherapy. Additionally, somatic mutation data were integrated, statistically analyzed, and visualized using the maftools R package (Mayakonda et al., 2018).

### SMR analysis

All analyses were conducted using the SMR software (https://yanglab. Westlake. Edu. cn/software/smr) (Zhu et al., 2016). GWAS data for LUSC were prepared, and the V8 release of GTEx eQTL/sQTL summary data was obtained for SMR analysis via CMD. The significance thresholds for the SMR analysis were set as pSMR <0.05 and pHEIDI >0.05.

### ST-seq analysis

Reverse compositional transcriptomic deconvolution facilitates the reverse calculation of the spatial distribution and relative abundance of individual cell types by analyzing the integrated gene expression profiles of whole tissues (Yan and Sun, 2023). In this study, this method was employed to infer cellular composition from spatial transcriptomics data. The mistyR package was used to explore spatial expression patterns of specified genes in tissues and analyze interactions between cell types across different spatial scales, aiming to reveal the construction mechanism of tissue functional units (Tanevski et al., 2022). A cell degree metric was applied to investigate interactions among distinct cell types, particularly for generating heterotypic scores. Spatial coordinate data were extracted using a scaling factor and converted into image coordinates, and the DBSCAN algorithm was used to construct a spatial adjacency network. The threshold for cell–cell interactions was set to 0.1, with parameters configured as nNeighbors = 6, sdist = 200, and maxdist = 200 to generate the adjacency matrix. Effective spatial connections were then filtered to obtain intercellular connection information (with minK = 4), and spatial distribution maps of cell interactions were generated. The nearest neighbor distances between specified cell types were quantitatively calculated using the STDistance package. Finally, the COMMOT method was used to infer the directionality of signal transduction based on the spatial distributions of ligands and receptors (Cang et al., 2023).

### Cell culture and transfection

Human acute monocytic leukemia cells (THP-1, Servicebio Biotech Co., Ltd., Wuhan, China), human normal lung epithelial cell line (BEAS-2B, Servicebio Biotech Co., Ltd., Wuhan, China), and two human lung squamous carcinoma cell lines (SK-MES-1 and NCI-H520, Pricella Biotech Co., Ltd., Wuhan, China) were cultured in Dulbecco’s Minimal Essential Medium (Invitrogen) supplemented with 10% fetal bovine serum (FBS, BI, Beit Haemek, Israel), 100 U/mL penicillin (BI), and 100 μg/mL streptomycin (BI). When THP-1 cells reached the exponential growth phase, 100 ng/mL phorbol 12-myristate 13-acetate (PMA, S1819, Beyotime Biotech Co., Ltd., Shanghai, China) was added to induce cell adherence and differentiation into M0 macrophages. Subsequently, M0 macrophages were polarized into the M2 phenotype through treatment using 20 ng/mL interleukin-4 (IL-4) (S1819, Beyotime Biotech Co., Ltd., Shanghai, China). All cells were cultured in a humidified incubator at 37 °C with 5% CO_2_. For the co-culture of M2 macrophages and tumor cells, SK-MES-1 cells were seeded in 6-well plates. After a period of growth, the conditioned medium collected from SK-MES-1 cultures was added to the culture medium of THP-1 cells. For functional validation assays involving midkine (MDK), MDK (P5727, Beyotime Biotech Co., Ltd., Shanghai, China) was added to the culture medium at a concentration of 20 ng/mL. Aiming to knockdown nucleolin (NCL) expression, three siNCL plasmids were used, while an sh-negative control plasmid served as the control. The control and siNCL plasmids were transfected into THP-1 cells using Invitrogen™ Lipofectamine™ 3,000 Transfection Reagent (Invitrogen Thermo Fisher Scientific, USA). The primer sequences used for plasmid construction are listed in Supplementary Table S2. For the induction of pyroptosis, when cell confluence reached approximately 70% after culture, lipopolysaccharide (LPS) was added at a final concentration of 1 μg/mL. After gentle mixing and continued incubation for 4 h, ATP solution was added at a final concentration of 5 mM to activate pyroptosis for an additional 0.5 h. The extent of pyroptosis was quantified by measuring lactate dehydrogenase (LDH) activity using a colorimetric assay kit (E-BC-K046-M, Elabscience Biotech Co., Ltd., Wuhan, China) to determine LDH activity in the cell culture supernatant.

### Animal experiments and sample collection

This study was conducted in accordance with the Declaration of Helsinki, and all animal experimental procedures were approved by the Laboratory Animal Ethics Committee of Beijing Friendship Hospital (Approval No. GENINK-20250,171). A total of 12 female BALB/c nude mice were used to establish a co-injection tumor model with tumor cells and M2 macrophages. The two groups of mice were inoculated with M2-polarized macrophages and M2 macrophages transfected with siNCL plasmid, respectively. Specifically, NCL-knockdown macrophages or NCL-normal macrophages (5 × 10^6^ cells/mouse) were mixed with 4 × 10^6^ SK-MES-1 cells and subcutaneously injected into nude mice. The experiment was terminated when mice reached humane endpoints (body weight loss exceeding 20% or tumor ulceration). Mice were euthanized, tumor tissues were collected for photographic documentation, and rapidly frozen for subsequent analysis.

### RNA extraction and quantitative real-time PCR (qPCR)

After the culture medium was discarded, the cells were washed and harvested, total cellular RNA was extracted using TRIzol reagent. Following reverse transcription, gene-specific primers were designed and synthesized (the primer sequences are listed in Supplementary Table S3). A reaction mixture containing cDNA, primers, and fluorescent dye was then prepared, and an amplification program was implemented. Gene expression levels were analyzed using a real-time fluorescence quantitative PCR instrument (CFX96 Touch, Bio-Rad Laboratories, Inc., California, USA).

### Enzyme-linked immunosorbent assay (ELISA)

The Human MDK ELISA Kit (E-EL-H6297) was purchased from Elabscience Biotechnology Co., Ltd. Following addition of the test sample, the plate was incubated and washed before the inclusion of the enzyme-labeled secondary antibody. The substrate was then added for color development. Absorbance was measured using an enzyme-labeled instrument, and the MDK concentration in the samples was calculated based on the standard curve.

### Immunofluorescence staining

Tumor tissue sections were deparaffinized and subjected to antigen retrieval by heating in citrate buffer (10 mM, pH 6.0) at 95 °C for 15 min. Subsequently, sections were blocked with 5% bovine serum albumin (BSA) in PBS for 1 h at room temperature, and then incubated with diluted primary antibodies overnight at 4 °C. After washing with PBS, sections were incubated with goat anti-rabbit secondary antibody (1:200 dilution) for 1 h at 37 °C. Nuclei were counterstained with 4′,6-diamidino-2-phenylindole (DAPI, 1 μg/mL, D9542, Sigma-Aldrich, USA) for 5 min, and autofluorescence quencher was added. The film was washed and sealed, and images were observed under a fluorescence microscope. Quantitative analysis of immunofluorescence images was performed using Saiviewer browsing and analysis software.

### Western blot analysis

Total proteins were extracted from the samples using RIPA buffer and quantified using the BCA Protein Assay Kit (CW0014, Cwbio, Taizhou, China). The protein samples were then separated by sodium dodecyl sulfate–polyacrylamide gel electrophoresis and transferred onto polyvinylidene fluoride membranes. After blocking, the membranes were incubated with primary antibodies, the details of which are listed in Supplementary Table S4. After washing, horseradish peroxidase-conjugated secondary antibodies were added. The membranes were subsequently washed again and developed using chemiluminescent reagents. Protein signals were captured using a gel imaging system, and the grayscale values of the protein bands were analyzed to determine relative protein expression levels.

### Statistical analysis

All statistical analyses were performed using R v4.2.1 and SPSS 20.0 (IBM, Armonk, NY, USA). Normality of continuous variables was tested using the Shapiro-Wilk test. For normally distributed data, two-group comparisons were performed using independent-sample t-test. For non-normally distributed data, Mann-Whitney U test or Kruskal–Wallis test was used. All statistical tests were two-sided, and P < 0.05 was considered statistically significant.

## Results

### Twelve gene expression modalities in PCD correlate with LUSC prognosis

**FIGURE 2:**
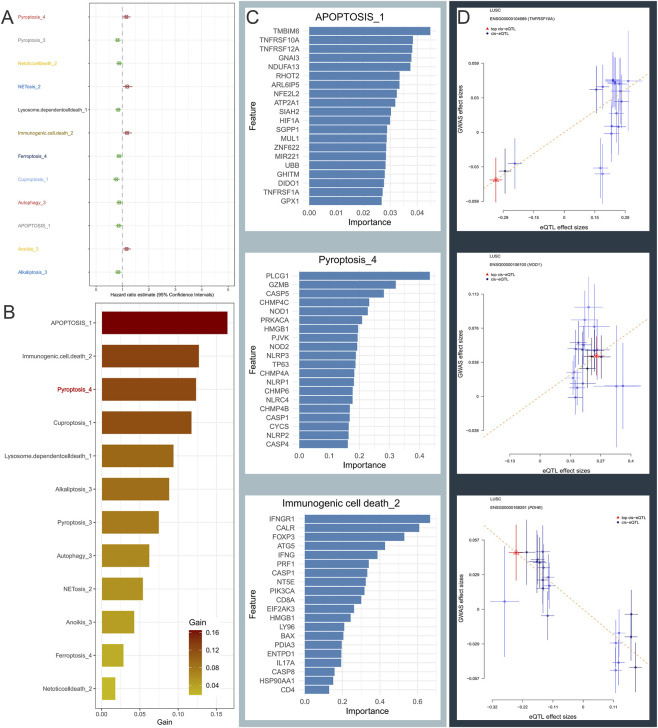
Multimodal autoencoder reveals prognosis-related PCD types. **(A)** Multiple prognosis-related PCD gene set signatures. Red indicates risk-associated factors, while green indicates protective factors. **(B)** Importance ranking of multiple prognosis-related PCD gene set signatures. **(C)** Top 20 genes ranked by importance within the top three most significant prognosis-related PCD gene set signatures. **(D)** SMR effect plot showing the relationship between altered expression of TNFRSF10A, NOD1, PDHB, and LUSC risk.

A multimodal autoencoder was used to extract 12 prognosis-related gene expression modality features from 18 PCD-associated gene sets, encompassing 11 PCD types (including pyroptosis, netotic cell death, lysosome-dependent cell death, immunogenic cell death, ferroptosis, and cuproptosis). Among these features, four were associated with poor prognosis in LUSC, whereas the remaining eight were related to favorable outcomes, demonstrating that PCD-related processes exhibit complex heterogeneity in the initiation and progression of LUSC. Notably, distinct genes within the same PCD pathway can spontaneously assemble into different modalities. For instance, pyroptosis features two distinct modalities (pyroptosis _3 and pyroptosis _4), exerting opposing effects on LUSC prognosis (Figure 2A). Ranking these modalities by their importance revealed the top three contributors: APOPTOSIS_1, Immunogenic cell death_2, and pyroptosis_4 (Figure 2B). Figure 2C shows the top 20 genes ranked by importance across the three modalities. Given that SMR analysis can assess whether SNP effects on phenotypes are mediated via gene expression, this method was employed to prioritize the screened genes. In the three aforementioned modalities, SMR identified pleiotropic or direct causal relationships between the altered expression of three genes (TNFRSF10A, NOD1, and PDHB) and LUSC risk. This finding strongly suggests that TNFRSF10 A and NOD1 may promote LUSC initiation and progression, whereas PDHB may exert an inhibitory effect (Figure 2D; Supplementary Figures S1A–C).

### NOD1 expression status on M2 macrophages influences pyroptosis scores and tumor microenvironment in LUSC

**FIGURE 3:**
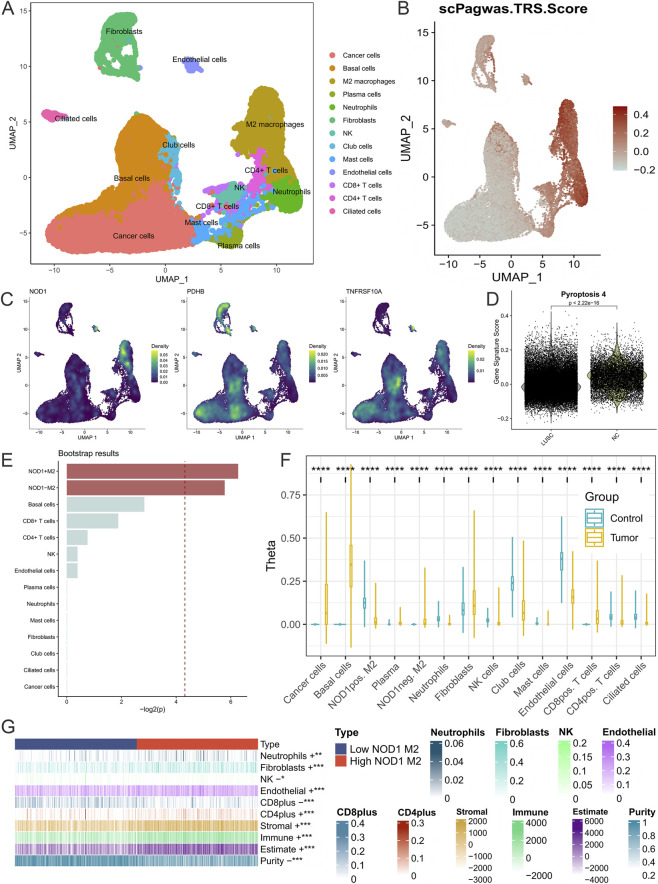
ScPagwas analysis reveals the critical role of M2 macrophages in LUSC and indicates that NOD1 and its representative pyroptosis alter the tumor microenvironment by influencing M2 macrophages. **(A)** UMAP plot showing the cell types present in LUSC. **(B)** TRS scores across various cell types obtained from scPagwas analysis, indicating their correlation with LUSC. **(C)** Expression patterns of NOD1, PDHB, and TNFRSF10 A across various cell types **(D)** Differences in Pyroptosis_4 gene set scores between LUSC and normal lung tissues. **(E)** Bootstrap results of scPagwas bias estimates for cell types associated with LUSC. **(F)** Differences in relative abundance of various cell types between LUSC and normal lung tissues. **(G)** Differences in immune microenvironment and ESTIMATE scores in LUSC between NOD1^+^ M2 and NOD1^−^ M2 macrophages. *P < 0.05, **P < 0.01, ***P < 0.001, ****P < 0.0001.

**FIGURE 4:**
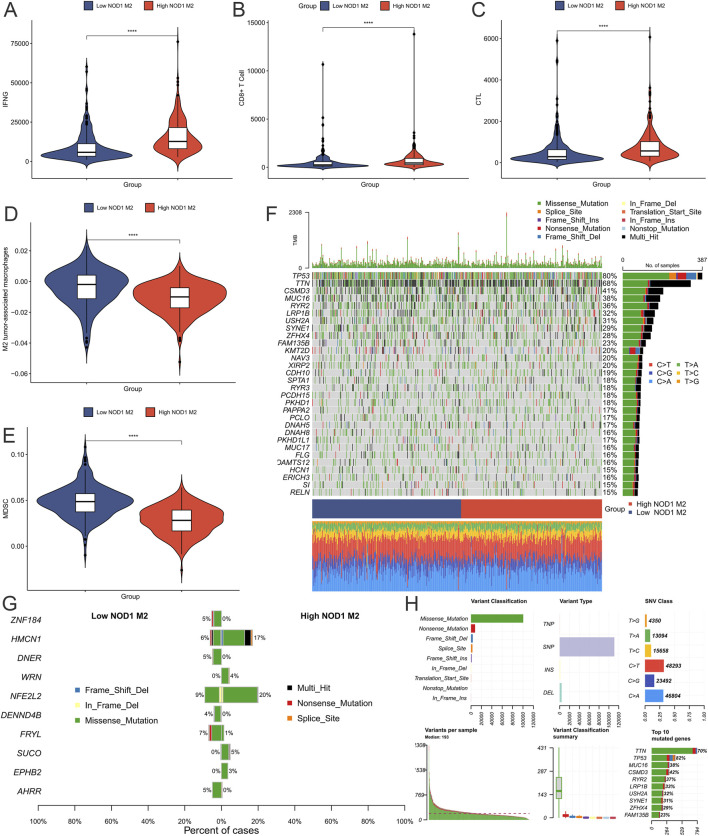
Expression level of NOD1 on M2 macrophages affects the tumor microenvironment of LUSC. **(A)** Difference in IFN-γ expression in LUSC between NOD1^+^ M2 and NOD1^−^ M2 macrophages. **(B)** Difference in relative abundance of CD8^+^ T cells in LUSC between NOD1^+^ M2 and NOD1^−^ M2 macrophages **(C)** Difference in relative abundance of CTLs in LUSC between NOD1^+^ M2 and NOD1^−^ M2 macrophages. **(D)** Difference in the relative abundance of M2 tumor-associated macrophages in LUSC between NOD1^+^ M2 and NOD1^−^ M2 macrophages. **(E)** Difference in relative abundance of myeloid-derived suppressor cells in LUSC between NOD1^+^ M2 and NOD1^−^ M2 macrophages. **(F)** Waterfall plot of somatic mutation characteristics in NOD1^+^ M2 and NOD1^−^ M2 macrophages; the bar chart on the right represents the proportion of each mutation type, and the numbers indicate the mutation frequency of each gene. **(G)** Heatmap of gene mutation frequency showing the mutation frequency and mutation type distribution of multiple genes across different sample groups. **(H)** MAF summary plot showing the mutational burden, mutation type distribution, and high-frequency mutated genes of samples; the number of mutations in the samples is shown as a stacked bar chart, and mutation types are represented as boxplots summarized by Variant_Classification. ****P < 0.0001.

After cell classification from 22 LUSC samples, 13 cell types were identified, including cancer cells, basal cells, M2 macrophages, and plasma cells (Figure 3A). ScPagwas analysis was performed to determine the LUSC-associated cell types. This method integrates pathway activity derived from scRNA-seq data with GWAS summary statistics to identify trait-relevant cell subsets. The results indicated that M2 macrophages had the strongest association with LUSC (Figure 3B). At the single-cell level, the expression levels of three key genes (TNFRSF10A, NOD1, and PDHB) were quantified. Among them, only NOD1 exhibited specific expression in M2 macrophages, and the pyroptosis _4 modal feature score associated with NOD1 differed significantly between LUSC and normal lung tissues (Figures 3C,D). This module was identified by the autoencoder from 1,964 PCD-related genes and consists of a gene set comprising 51 pyroptosis-related genes, whose expression signature was significantly negatively correlated with the prognosis of LUSC patients (the complete list of genes is provided in Supplementary Material Table S5). Bootstrap bias estimation further supported a strong association between NOD1^+^ M2 macrophages, NOD1^−^ M2 macrophages, and LUSC (Figure 3E). Given that NOD1 expression is highly enriched in M2 macrophages in LUSC tissues and is a well-established key regulator of the pyroptosis pathway, whereas TNFRSF10 A and PDHB are broadly expressed across multiple cell types with low cell-type specificity and their functions are primarily associated with apoptosis and immunogenic cell death, respectively—both showing limited relevance to the pyroptosis phenotype of interest in this study. Therefore, NOD1, M2 macrophages, and pyroptosis were selected for further investigation. Significant differences in infiltration levels were observed between tumor and normal lung tissues for NOD1^+^ and NOD1^−^ M2 macrophages, with a marked increase in the abundance of NOD1^−^ M2 macrophages in tumors (Figure 3F). To elucidate the association between NOD1 expression and established M2 macrophage subclusters, M2 macrophages were subjected to re-clustering and fine characterization, leading to the identification of three functionally heterogeneous subclusters (M2a, M2b, and M2c). The M2a subcluster exhibited high expression of genes associated with immunosuppression and lipid metabolism reprogramming (*SPP1*, *CSTB*, *PLIN2*), suggesting a role in promoting tumor immune evasion. The M2b subcluster showed high expression of genes involved in immunomodulation, inflammatory regulation, angiogenesis, and tissue remodeling (*S100A8*, *FPR1*, *H3F3B*), displaying functional features associated with pro-tumor metastasis. The M2c subcluster demonstrated high expression of genes related to anti-inflammatory responses, immune tolerance, and iron metabolism (*SLC40A1*, *C1QA*, and *C1QB*), participating in complement-mediated immune regulation (Supplementary Figures S3A,B). Notably, the M2b subcluster almost completely overlapped with NOD1^-^M2 macrophages, indicating the specificity of NOD1 as a negative marker for this functional subcluster in the context of LUSC (Supplementary Figure S3C). To determine whether the M2b subcluster and NOD1^-^M2 macrophages also exhibited functional consistency, functional heterogeneity validation was performed for both. GO enrichment analysis revealed that the M2b subcluster was distinguished from the M2a and M2c subclusters primarily by regulation of cell-cell adhesion, chemotaxis, enzyme inhibitor activity, amide binding, and peptide binding, and these functional differences were also reflected in the comparison between NOD1^-^M2 and NOD1^+^M2 macrophages (Supplementary Figure S3D). KEGG analysis indicated that the major signaling differences between the M2b subcluster and the M2a/M2c subclusters involved lysosome biogenesis, complement and coagulation cascades, apoptosis, phagosome, and antigen processing and presentation, and these signaling differences were also observed between NOD1^-^M2 and NOD1^+^M2 macrophages. More importantly, differentially expressed genes between the M2b and M2a subclusters were directly enriched in the NOD-like receptor signaling pathway and apoptosis, further confirming that differences in NOD1 expression can mediate macrophage pyroptosis-like cell death and anti-tumor immune activation (Supplementary Figure S2E). Furthermore, the tissue microenvironment differed significantly between tumor samples predominantly containing NOD1^+^ M2 macrophages and those dominated by NOD1^−^ M2 macrophages. The NOD1^−^ M2 macrophage group had lower ESTIMATE immune and scores and neutrophil infiltration, along with higher tumor purity (Figure 3G). TIDE-based immunotherapy analysis further validated these findings. In terms of antitumor immunity, the NOD1^−^ M2 macrophage group exhibited decreased IFN-γ expression and reduced infiltration of cytotoxic T lymphocytes (e.g., CD8^+^ T cells), accompanied by high levels of M2 tumor-associated macrophages and myeloid-derived suppressor cells. The findings indicate that patients in the NOD1^−^ M2 macrophage group may be less likely to benefit from immunotherapy (Figures 4A–E). Additionally, NOD1 expression in M2 macrophages may be correlated with tumor mutational burden. For example, the HMCN1 demonstrated a 17% mutation rate in the NOD1^+^ M2 macrophage group, primarily comprising missense mutations, whereas the mutation rate in the NOD1^−^ group was 8%, including missense mutations and frameshift deletions. Overall, the NOD1^−^ M2 macrophage group had a lower mutational burden, indicating weakened genomic instability and potentially reduced sensitivity to immunotherapy in this tumor subtype (Figures 4F–H).

### Prognostic model based on Pyroptosis_4 gene expression signature exhibits predictive efficacy in early-stage LUSC

**FIGURE 5:**
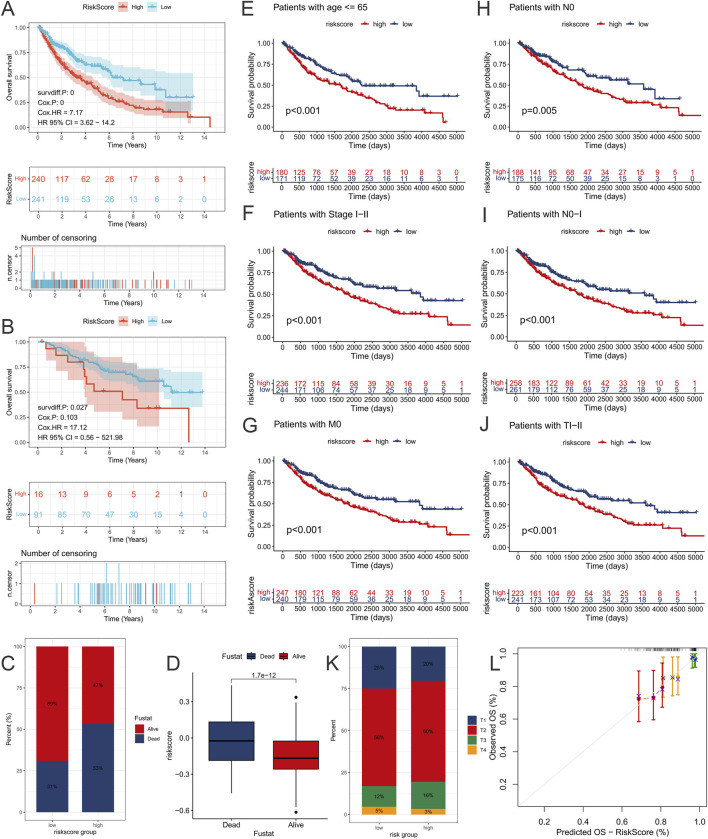
Predictive efficacy and comprehensive evaluation of the prognostic model. **(A)** Comparison of survival status between high- and low-risk groups in the training set. **(B)** Comparison of survival status between high- and low-risk groups in the validation set. **(C)** Proportion of patients with different outcomes in high- and low-risk groups. **(D)** Significant differences in risk scores were observed between patients with different outcomes. **(E–J)** Survival differences between high- and low-risk groups in six early-stage subgroups: age ≤65 years, stage I–II, M0, N0, N0–1, and T I–II. **(K)** Distribution of patients at T1, T2, T3, and T4 stages in high- and low-risk groups. **(L)** Calibration curves of the model demonstrate the correlation between risk scores and predicted overall survival. Green, orange, and red represent calibration curves for 1-year, 3-year, and 5-year survival rates, respectively.

Aiming to clarify the roles of NOD1, M2 macrophages, and pyroptosis in the prognosis of LUSC, a neural network-based prognostic prediction model was constructed using data from the TCGA-LUSC cohort. This model stratified patients into high- and low-risk groups. Kaplan–Meier survival curves showed that high-risk patients had significantly shorter overall survival than low-risk patients (P < 0.05, Figure 5A). The classification performance of the model was further validated using an external validation set. Survival analysis revealed that the low-risk group had a markedly better prognosis than the high-risk group (P < 0.05, Figure 5B; Supplementary Figure S2A), confirming model stability. High-risk patients generally faced a higher mortality risk overall (Figures 5C,D). Subgroup analysis revealed that the model had a favorable prognostic value in early-stage patient populations. In subgroups of patients aged ≤65 years, at stages I–II, M0, N0, or N0-1, the high-risk group consistently showed shorter survival rates (Figures 5E–I). Similarly, a comparable trend was observed in patients at T I–II stage: the proportion of T1 stage patients in the low-risk group was significantly higher than that in the high-risk group (25% vs. 20%; Figures 5J,K). The calibration curves of the model indicated a significant negative correlation between the risk score and predicted overall survival, with the risk score demonstrating the strongest robustness at the early stage (1 year, Figure 5L; Supplementary Figure S2B). SHAP quantified the contribution of each gene in the model to tumor prognosis, indicating that IL-6, NOD1, GSDMD, and other genes are core drivers of tumor prognosis (Supplementary Figure S2C). Supplementary Figure S2D shows the distribution of clinical characteristics and key genes across different risk groups, revealing elevated expressions of the pro-inflammatory genes CASP4, CASP9, IL-1β, and IL-6 in the high-risk group. Immune microenvironment analysis validated this finding: immune activities, including parainflammation and co-stimulation, were upregulated in the high-risk group, indicating a prominent pro-inflammatory state (Supplementary Figure S2E). Finally, the infiltration proportion of NOD1^-^M2 macrophages in the high-risk group was higher than that in the low-risk group, suggesting a correlation between the prognostic model and the functional status of M2 macrophages (Supplementary Figure S2F).

### NOD1^−^ M2 macrophages form a proximal spatial network with cancer cells

**FIGURE 6:**
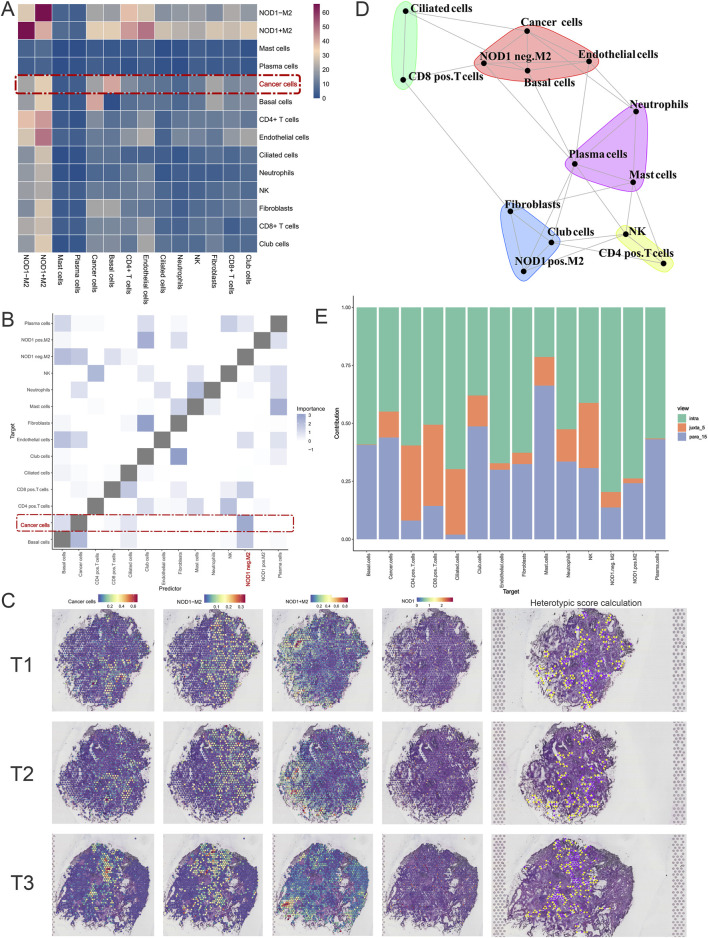
Spatial interaction relationship between NOD1- M2 macrophages and lung squamous cell carcinoma cells. **(A)** ScRNA-seq reveals the communication intensity between cancer cells and other cell types. **(B)** ST-seq analysis reveals the communication intensity between cancer cells and other cell types. **(C)** Spatial distribution of key genes and cell types within tumors. The heterotypic cell network reveals the spatial interaction pattern between NOD1^-^ M2 macrophages and cancer cells, where yellow represents NOD1^-^ M2 macrophages and purple represents cancer cells. **(D)** Interaction clusters among various cell types from an internal perspective. **(E)** Contribution of communication modes at different spatial distances to the intercellular communication intensity among various cell types.

The spatial distribution of immune cells within tumors is highly heterogeneous, a feature that supports their specific intercellular communications. Aiming to explore whether NOD1 expression mediates changes in the spatial distribution of M2 macrophages and its impact on their intercellular communication, scRNA-seq and ST-seq analyses were conducted. ScRNA-seq analysis confirmed that NOD1^+^ M2 and NOD1^-^ M2 macrophages showed substantial differences in the intensity of intercellular communication with tumor cells, indicating the potential involvement of NOD1 expression on M2 macrophages (Figure 6A). ST-seq further revealed that among all other cells in the tumor microenvironment (including immune cells, fibroblasts, endothelial cells), NOD1^-^ M2 macrophages maintained the closest intercellular communication with tumor cells (Figure 6B). Spatially, NOD1^-^ M2 macrophages overlapped extensively with tumor cells, whereas the opposite pattern was observed for NOD1^+^ M2 macrophages. The expression distribution pattern of NOD1 closely corresponded to the infiltration pattern of NOD1^+^ M2 macrophages. Aiming to further assess whether NOD1^-^ M2 macrophages form a cellular regional “networks” with tumor cells, their spatial adjacency patterns were analyzed. The results showed that NOD1^-^ M2 macrophages were consistently associated with tumor cells and tended to surround them (Figure 6C). This preference for proximal intercellular communication was further supported by mistyR analysis. From the internal perspective based on marker expression within individual spots, NOD1^-^ M2 macrophages showed a close relationship with tumor cells (Figure 6D), and this internal perspective played a dominant role in intercellular communication (Figure 6E). Quantitative analysis of the nearest neighbor distance between cells ultimately confirmed this finding, namely, that NOD1^-^M2 macrophages exhibited a significantly shorter spatial distance to tumor cells compared with NOD1^+^M2 macrophages (Supplementary Material Figures S4A–F). Overall, these results indicate that cancer cells and NOD1^-^ M2 macrophages preferentially engage in proximal intercellular communication.

### Cancer cells inhibit pyroptosis of M2 macrophages via the MDK/NCL pathway

**FIGURE 7:**
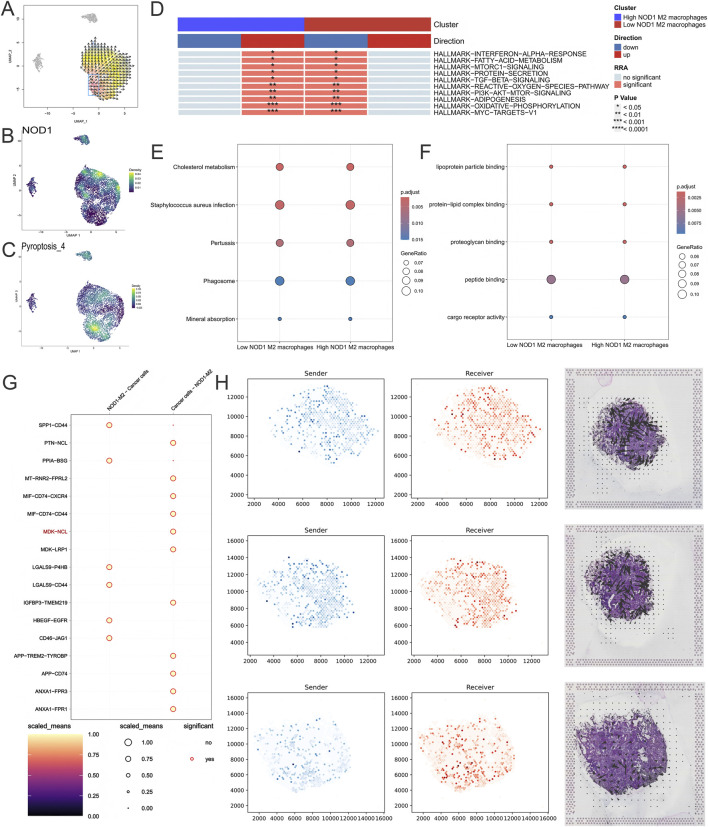
Effects of NOD1 on pyroptosis of M2 macrophages and their intercellular communication with lung squamous cell carcinoma cells. **(A)** Pseudotime analysis reveals the developmental origin and differentiation trajectory of M2 macrophages. **(B)** Heterogeneity of NOD1 expression levels in M2 macrophages at different stages. **(C)** Pyroptosis_4 gene set scores of M2 macrophages across different stages. **(D)** GSVA enrichment analysis of differentially expressed genes between M2 macrophages with high and low NOD1 expression. **(E)** KEGG pathway enrichment analysis of differentially expressed genes between M2 macrophages with high and low NOD1 expression. **(F)** GO enrichment analysis of differentially expressed genes between M2 macrophages with high and low NOD1 expression. **(G)** Activation status of key ligand-receptor pairs under different communication patterns between M2 macrophages and tumor cells. **(H)** Spatial distribution of signal sending/receiving volumes of the ligand-receptor pair (MDK/NCL). Blue scatter plots denote signal-sending cells (expressing ligand MDK), red scatter plots represent signal-receiving cells (expressing receptor NCL), with darker colors indicating stronger signals. Arrows on the tissue sections represent the signal flow direction, and more prominent arrows indicate increased dependence on MDK/NCL for intercellular communication.

**FIGURE 8:**
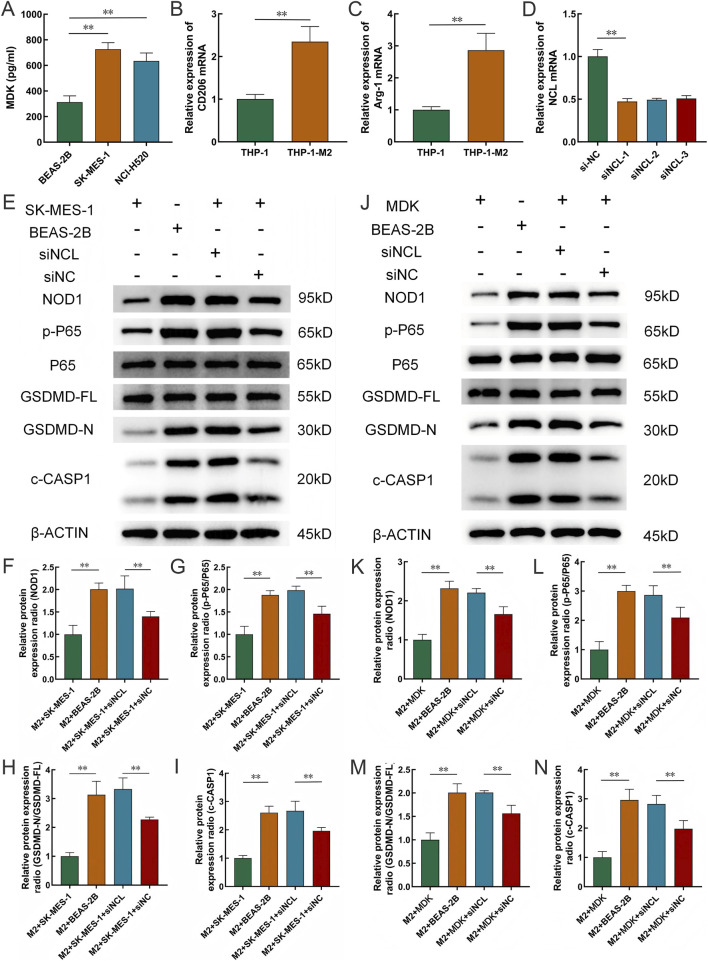
Effects of lung squamous cell carcinoma cells and MDK on the pyroptosis process of M2 macrophages. **(A)** Concentration of MDK secreted by different cell lines. **(B)** Expression level of CD206 mRNA in macrophages after induced polarization. **(C)** Expression level of Arg-1 mRNA in macrophages after induced polarization. **(D)** Comparison of knockdown efficiency among three plasmids. **(E)** Expression of pyroptosis-related proteins after co-culture with the SK-MES-1 cell line and NCL knockdown. **(F)** Expression levels of NOD1 protein under different treatment conditions. **(G)** Expression levels of p-P65 protein under different treatment conditions. **(H)** Expression levels of GSDMD-N protein under different treatment conditions. **(I)** Expression levels of c-CASP1 protein under different treatment conditions. **(J)** Expression of pyroptosis-related proteins after MDK supplementation and NCL knockdown. **(K)** Expression levels of NOD1 protein under different treatment conditions **(L)** Expression levels of p-P65 protein under different treatment conditions **(M)** Expression levels of GSDMD-N protein under different treatment conditions **(N)** Expression levels of c-CASP1 protein under different treatment conditions.

**FIGURE 9:**
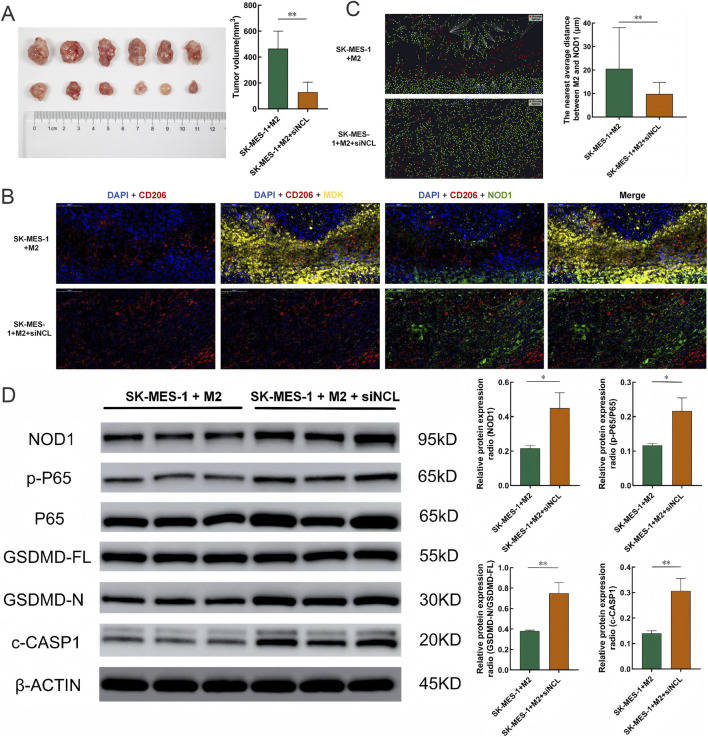
*In vivo* validation of the MDK/NCL axis in a LUSC co-injection mouse model. **(A)** Representative images of subcutaneous tumors and quantitative analysis of tumor volume in the SK-MES-1 + M2 group and the SK-MES-1 + M2 + siNCL group. Scale bar: cm. **(B)** Multiplex immunofluorescence staining of tumor tissues for DAPI (blue), CD206 (red), MDK (yellow), and NOD1 (green). **(C)** Representative images of spatial distribution (left) and quantitative analysis of the nearest average distance between M2 macrophages (CD206^+^) and NOD1^+^cells (right). **(D)** Western blot analysis (left) and quantification (right) of NOD1, p-P65, total P65, GSDMD-FL, GSDMD-N, and cleaved caspase-1 (c-CASP1) protein expression in tumor tissues. *P < 0.05, **P < 0.01.

NOD1 is a critical gene that promotes pyroptosis (Qu et al., 2025). Given that the expression level of NOD1 on M2 macrophages correlates with their spatial distance from tumor cells, this study hypothesized that tumor cells regulate NOD1 expression on M2 macrophages through ligand-receptor binding, a common short-range communication mechanism. This process protects M2 macrophages from pyroptosis during development and differentiation, allowing tumor cells to evade immune surveillance and elimination, thus further contributing to tumor progression. Pseudotime analysis confirmed that M2 macrophages originated from a common initial differentiation point (Figure 7A). During M2 macrophage differentiation, as NOD1 expression gradually increased, the score of the pyroptosis _4 gene set markedly changed, indicating that NOD1 expression modulates pyroptosis in M2 macrophages (Figures 7BC). Functional enrichment analysis revealed differences in receptor activity and phagocytic function between M2 macrophages with high and low NOD1 expression levels. M2 macrophages with high NOD1 expression showed more robust inflammatory pathway activation and reactive oxygen species release. This finding indicates that NOD1 expression may promote pyroptosis of M2 macrophages in LUSC, which is consistent with previous reports (Figure 7D–F) (Qu et al., 2025). Ligand-receptor pair analysis of intercellular communication identified MDK and NCL as key ligands and receptors, respectively, and were thus repeatedly enriched. MDK exhibits the characteristics of a ligand protein, and the MDK/NCL axis is a signaling pathway that mediates interactions between M2 macrophages and tumor cells. Importantly, this pathway was only activated when tumor cells acted as ligand releasers, but not in the reverse direction, indicating a fixed signal transduction orientation (Figure 7G). COMMOT analysis based on ST-seq further visualized the spatial communication signal flow of the MDK/NCL pathway, confirming its flow from tumor cells to M2 macrophages rather than *vice versa*, indicating that this pathway may mediate tumor cell regulation of M2 macrophage pyroptosis (Figure 7H). Experimental studies were also performed to validate these findings. ELISA demonstrated that compared with the BEAS-2B cell line (human normal lung epithelial cells), the concentration of MDK in the culture supernatants of two human lung squamous carcinoma cell lines (SK-MES-1 and NCI-H520) increased by more than 1-fold, indicating that LUSC cells secrete MDK into the tumor microenvironment (Figure 8A). Among these cell lines, SK-MES-1 exhibited the highest MDK secretion level. Subsequently, after inducing THP-1 cell differentiation with phorbol PMA and IL-4, qPCR results confirmed the upregulated expression of CD206 mRNA and Arg-1 mRNA, verifying the successful polarization of M0 macrophages into the M2 phenotype (Figures 8B,C). Co-culture of M2 macrophages with SK-MES-1 cell-conditioned medium significantly inhibited NOD1 expression in M2 macrophages. Conversely, co-culture had no effect on the expression of full-length GSDMD (GSDMD-FL) protein but markedly downregulated the expression of its active fragment GSDMD-N and inflammatory proteins p-p65 and Cleaved Caspase1. PCR analysis also showed reduced expression of pyroptosis-related inflammatory mediators, including TNF-α, IL-1β, IL-18, CXCL-10, and MCP-1 mRNA, indicating that lung squamous carcinoma cells suppress pyroptosis-associated pro-inflammatory responses and cell death functions in M2 macrophages. Aiming to examine whether this effect depended on the MDK/NCL signaling pathway, plasmids targeting NCL knockdown in M2 macrophages were constructed. Among the three plasmids, siNCL-1 exhibited the most efficient knockdown and was therefore used for subsequent experiments (Figure 8D). After NCL knockdown, the aforementioned inhibitory effects were reversed, indicating that the suppression of M2 macrophage pyroptosis by lung squamous carcinoma cells is dependent on NCL (Figure 8E–I). Aiming to confirm whether this effect was mediated by MDK secreted by lung squamous carcinoma cells, the above experiments were repeated using recombinant human MDK protein. The results showed that MDK alone could inhibit pyroptosis (marked by NOD1 and GSDMD-N expression) and inflammatory response (marked by Cleaved Caspase1 and IL-1β mRNA expression) in M2 macrophages (Figure 8J–N). LDH release assays also demonstrated that both supplementation of MDK in the culture medium and co-culture with tumor cells suppressed LDH release following pyroptosis in M2 macrophages (Supplementary Figures S5A–K). However, these effects were eliminated after NCL knockdown, confirming the occurrence of signal transduction between MDK and NCL and the inhibition of M2 macrophage pyroptosis by LSCC cells through the MDK/NCL pathway. Subsequent *in vivo* studies validated this conclusion. Compared with subcutaneous co-injection of tumor cells and M2 macrophages, tumor volume was significantly reduced upon knockdown of NCL expression in M2 macrophages (Figure 9A). Multiplex immunofluorescence staining revealed that, in the nude mouse model co-injected with M2 macrophages and tumor cells, MDK protein was significantly enriched at the membrane and in the cytoplasm of M2 macrophages. In contrast, in the nude mouse model with NCL knockdown in M2 macrophages, the concentration of MDK protein surrounding M2 macrophages was markedly decreased, suggesting that the absence of NCL precludes the establishment of intact ligand-receptor interactions, thereby impairing the ability of tumor cell-secreted MDK to effectively act on M2 macrophages (Figure 9B). Furthermore, in the nude mouse model co-injected with M2 macrophages and tumor cells, NOD1 protein expression was not only lower, but also exhibited a significantly greater average spatial distance from M2 macrophages (Figure 9C). Quantitative analysis showed that *in vivo* NCL knockdown also led to significant upregulation of pyroptosis-related proteins, including GSDMD-N and NOD1, as well as inflammatory proteins such as p-p65 and c-CASP1 (Figure 9D), consistent with the *in vitro* findings, suggesting that M2 macrophages are protected from pyroptosis through this pathway, thereby promoting the formation of an immunosuppressive microenvironment.

## Discussion

Numerous studies have demonstrated that, from the perspective of cell fate, dysregulation of various types of PCD plays an indispensable role in tumorigenesis and progression (Li et al., 2024; Liu et al., 2024; Gao et al., 2024; Kashefizadeh and Kazemizadeh, 2023; Jiang et al., 2026). However, tumors encompass diverse cellular interactions that are shaped by specific spatial distribution patterns. Analyses limited to a single type of PCD or a single cell population fails to capture the holistic effects of multi-gene and intercellular collaboration. Therefore, by integrating bulk RNA-seq, scRNA-seq, and ST-seq data with deep learning and summary-data-based SMR approaches, this study elucidated the specific mechanism by which NOD1 mediates altered pyroptosis in M2 macrophages to sustain an immunosuppressive microenvironment in LUSC. For LUSC, the key PCD modalities associated with prognosis primarily include apoptosis, immunogenic cell death, and pyroptosis. Among these, pyroptosis holds unique prognostic relevance and is mediated by the NOD1 gene. In collaboration with M2 macrophages—the cell type most strongly associated with LUSC traits—NOD1 contributes to shaping the activation state of the tumor immune microenvironment. With the MDK/NCL pathway as a link, tumor cells transmit MDK-derived signals to M2 macrophages within a tightly connected spatial network formed, preserving their pro-tumorigenic capacity by inhibiting pyroptosis. Additionally, an effective and robust prognostic model was constructed, and its predictive performance was validated in independent datasets. Patient stratification based on this model yielded remarkable benefits even in early-stage LUSC, with high-risk patients demonstrating a poorer prognosis. Overall, these findings provide novel insights into the precision treatment of LUSC.

In this study, the analytical results of the multimodal autoencoder expanded the cognitive dimensions of PCD in LUSC, revealing that different genetic modalities within the same PCD type can exhibit completely opposite prognostic effects. This heterogeneity may stem from the differential activation of PCD pathways across distinct cell subsets. Activation of the pyroptosis pathway in tumor cells induces immunogenic cell death, and the released tumor antigens promote dendritic cell maturation, further enhancing anti-tumor immune response. Nanoreactors developed based on this principle have been shown to improve the efficacy of immunotherapy for NSCLC (Sun et al., 2025; Xu et al., 2026). Conversely, inhibition of the pyroptosis pathway in M2 macrophages reduces the release of pro-inflammatory cytokines, impairs the anti-tumor capacity of natural killer cells, and accelerates tumor progression. Meanwhile, initiation of inflammatory and cell death programs in T cells may promote tumorigenesis (Wang et al., 2025; Patton et al., 2025). Similarly, although Mendelian randomization provides robust genetic evidence supporting a potential causal relationship between NOD1 and increased LUSC risk, subsequent experiment results have indicated that NOD1 mediates pyroptosis in M2 macrophages. This phenomenon may also be linked to the expression pattern of NOD1 in LUSC. While NOD1 is primarily expressed in macrophages, its activation or inhibition in other cell subsets can trigger entirely opposite effects, ultimately resulting in macroscopic differences in tumorigenesis and survival outcomes. Collectively, these findings indicate that tumor treatment requires precise differentiation of cell- and pathway-specific PCD rather than a generalized assessment of the overall PCD status. In future clinical practice, single-cell sequencing technology can be employed to further characterize the activation status of PCD pathways across different cell subsets, providing highly granular evidence for the formulation of personalized treatment regimens.

Targeting macrophages to disrupt tumor immune tolerance and activate host immune defense represents the next research direction in cancer immunotherapy. The MDK/NCL axis, a key signaling pathway, was previously well-characterized for mediating crosstalk between cancer-associated fibroblasts and other immune cells in triple-negative breast cancer, neuroblastoma, and lung adenocarcinoma; however, its role in macrophages remains underexplored (Ma et al., 2025; Chu et al., 2025; Du et al., 2023). Recent studies have shown that in colorectal cancer, MDK-expressing tumor cells colocalize with NCL-expressing macrophages within an immunosuppressive microenvironment driven by MDK/NCL pathway-mediated shifts in macrophage polarization states (Liu et al., 2025). In lung adenocarcinoma, high MDK/NCL expression is associated with reduced immune cell infiltration. Patients with elevated MDK/NCL levels also exhibit upregulated immune checkpoint genes, including PD-1 and CTLA-4, and poorer survival, indicating the potential role of this pathway in mediating immune checkpoint inhibitor resistance, tumor progression, and immune evasion (Fu et al., 2025). In LUSC, this study is the first to link the MDK/NCL axis to altered pyroptotic activity in M2 macrophages. The results revealed that downregulation in M2 macrophages inhibited the activation of key pyroptotic pathway molecules (e.g., GSDMD-N, Cleaved Caspase-1) and reduced the release of inflammatory cytokines. Spatial transcriptomic analysis further confirmed that NOD1^-^ M2 macrophages form a tight spatial interaction network with tumor cells, maintaining an immunosuppressive state via close-range communication. This mechanism explains why LUSC patients enriched with NOD1^-^ M2 macrophages show poor responses to immunotherapy. The resulting immunosuppressive microenvironment not only inhibits cytotoxic T-cell infiltration and activation but also diminishes tumor cell immunogenicity. These findings support the hypothesis that modulating PCD pathways could restore antitumor immunity in LUSC and enhance treatment responses. Although MDK and NCL interact with high affinity, requiring the development of highly specific inhibitors, tumor cells may compensate for MDK/NCL pathway inhibition by activating alternative resistance mechanisms. However, structural biology-driven analysis of the MDK-NCL binding mode offers a potential therapeutic approach. Specifically, activation of the NOD1 pathway in M2 macrophages to restore pyroptosis and remodel the tumor immune microenvironment may provide a novel therapeutic strategy for LUSC.

Despite these findings, this study has several limitations. First, the samples were primarily derived from European populations; therefore, caution is warranted when extrapolating the conclusions to other ethnic groups. Second, the number of spatial transcriptomics samples was small, and the spatial interactions between tumor cells and NOD1^−^ M2 macrophages require further verification using additional tissue sections. Third, although both *in vitro* and *in vivo* studies have validated the role of the MDK/NCL pathway, the therapeutic value of certain strategies with translational potential, such as MDK inhibitors, remains limited by the lack of appropriate targeted delivery approaches and thus still requires *in vivo* animal model validation. Finally, the interactive regulatory mechanisms among different PCD modalities warrant further exploration. In the future, multicenter, cross-ethnic clinical cohort studies should be conducted, and the therapeutic efficacy of MDK/NCL pathway inhibitors should be validated *in vivo*.

## Conclusion

Overall, through integrated multi-omics analyses, this study highlights the central role of pyroptosis as a core PCD type in the prognosis and immune regulation of LUSC. Differential expression of NOD1 in M2 macrophages contributes to shaping the tumor immune microenvironment, while tumor cells form a spatial interaction network with M2 macrophages via the MDK/NCL pathway to modulate pyroptosis. These findings not only advance the understanding of PCD heterogeneity in LUSC but also identify novel targets to overcome these therapeutic limitations. Targeting the MDK/NCL pathway, particularly in combination with existing immune checkpoint inhibitors, may represent a promising strategy for improving survival outcomes in LUSC.

## Data Availability

The datasets presented in this study can be found in online repositories. The names of the repository/repositories and accession number(s) can be found in the article/Supplementary Material.
