# Supplementary material for: Integrative multi-omics and mendelian randomization reveal the critical role of pyroptosis in prognosis and therapy of lung squamous cell carcinomas
Source: Front Cell Dev Biol. 2026 Jul 8;14:1822831. doi: 10.3389/fcell.2026.1822831 (PMC13388469; doi:10.3389/fcell.2026.1822831)
Supplement: Supplementary file 2 [file DataSheet1.docx]

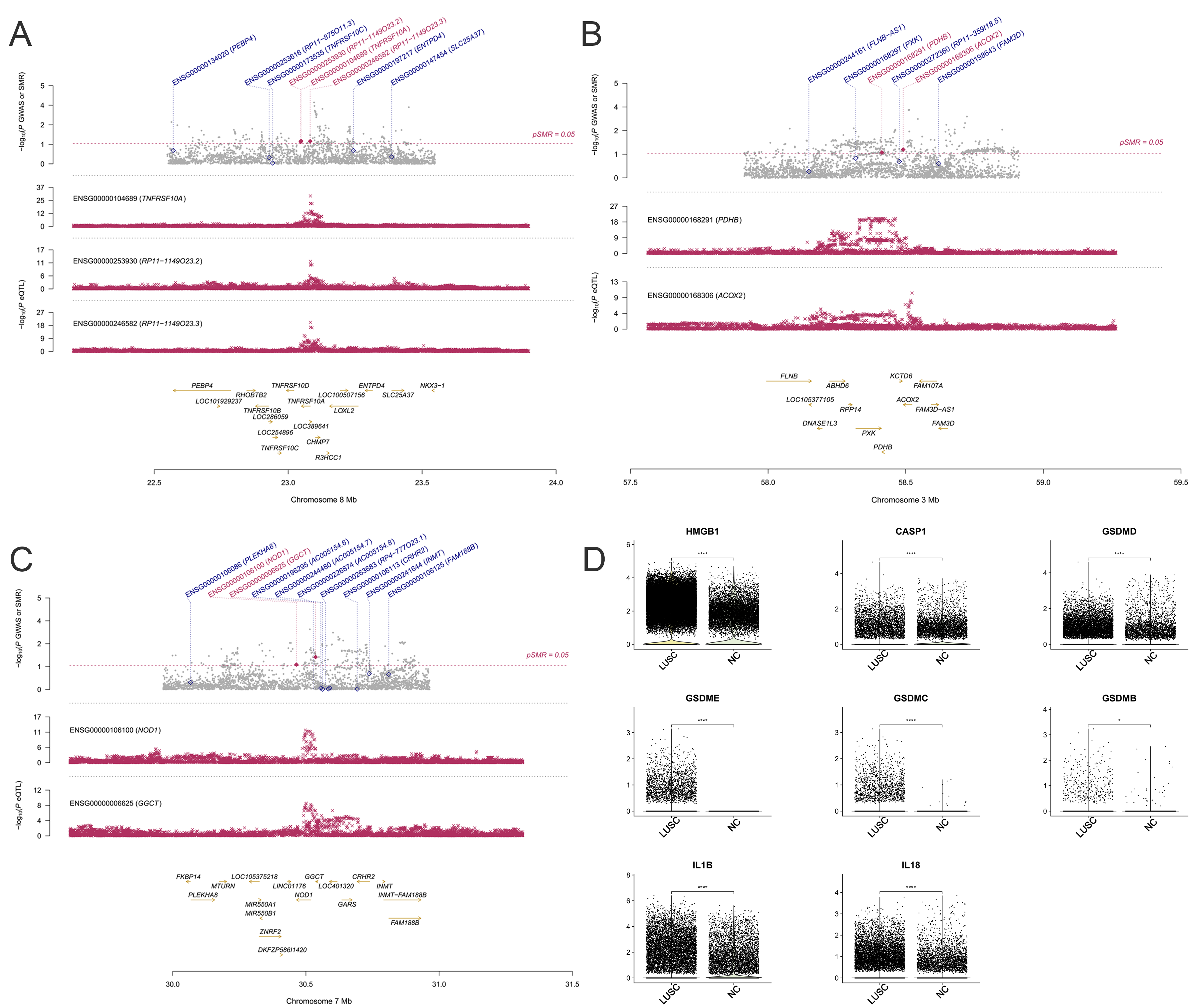


**Fig. S1 Localization and Expression of Key Genes.** (A-C) SMRLocusPlot illustrates the causal associations between the expression of TNFRSF10A, NOD1, and PDHB genes and LUSC within specific genomic regions, as well as which SNPs regulate the expression of these genes, along with the genomic positions of these genes and their neighboring genes. The three main panels from top to bottom are the Manhattan plot, expression quantitative trait locus (eQTL) scatter plot, and gene annotation plot. The red dashed line represents the significance threshold of p_SMR = 0.05, points above this line indicate statistically significant associations. Peak genes in the Manhattan plot suggest that their expression levels may influence phenotypes via the SNPs they regulate, implying potential causal relationships. Peak genes in the eQTL scatter plot indicate that the corresponding SNP exerts a strong regulatory effect on gene expression. SNPs with high eQTL signals are cis-regulatory loci, i.e., genetic variations located near the target gene (typically <1 Mb) that modulate its expression. (D) Single-cell sequencing reveals the expression differences of typical pyroptosis-related genes between LUSC and normal lung tissues.


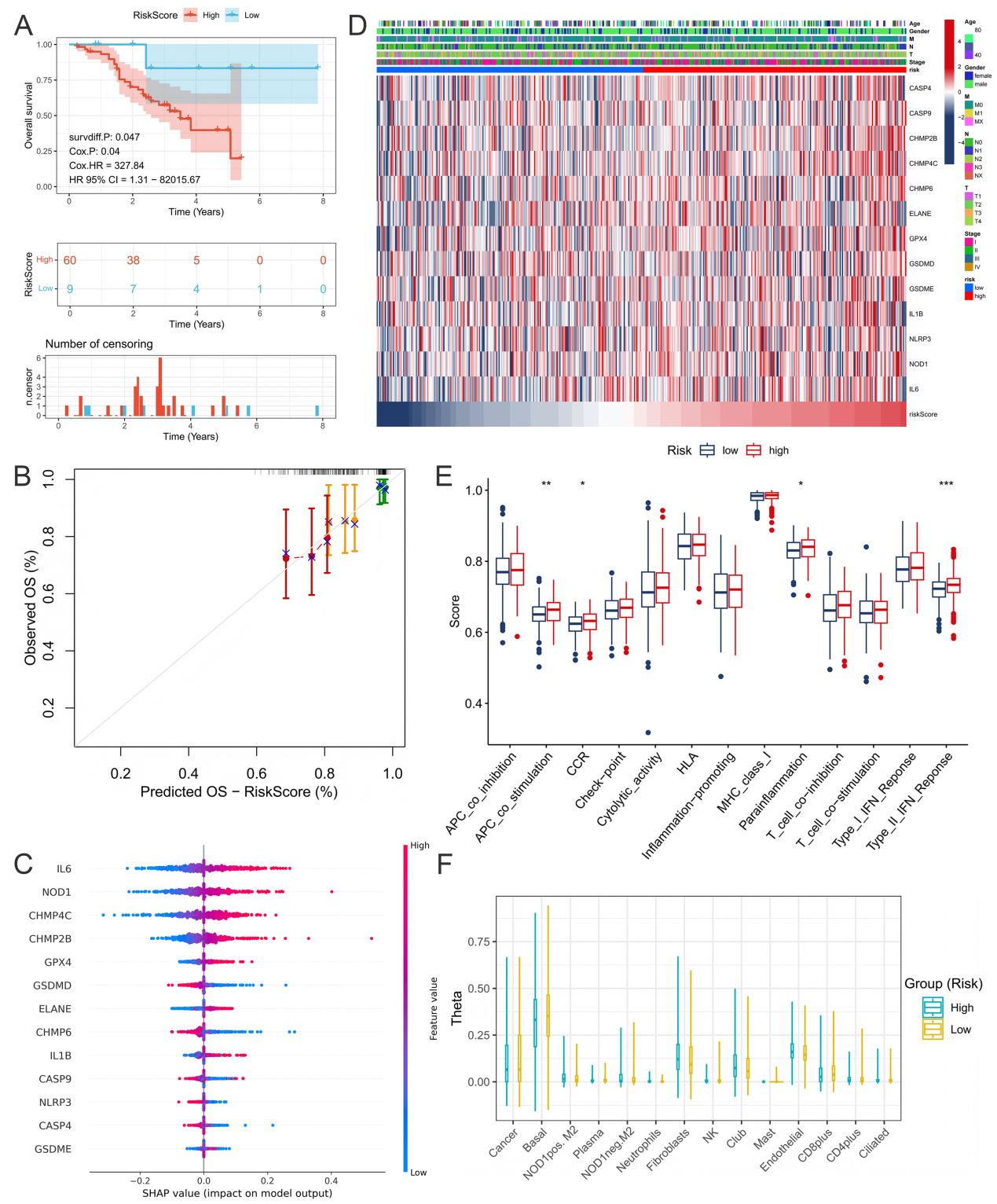


**Fig. S2 Quantitative Analysis of the Prediction Model and Its Association with the Immune Microenvironment of LUSC.** (A) Comparison of survival status between high- and low-risk groups in the validation set. (B) Calibration curves of the model demonstrate the correlation between risk scores and predicted overall survival. Green, orange, and red represent calibration curves for 1-year, 3-year, and 5-year survival rates, respectively. (C) The summary plot displays the importance ranking of genes constituting the prediction model and their impact on prognosis. The Y-axis shows genes sorted by feature importance, with genes positioned higher having a greater influence on model predictions. The X-axis represents SHAP values, which indicate the contribution of each feature to the model output. The "scatter cloud" for each gene illustrates the distribution of SHAP values for that gene across all samples. (D) Differences in the expression status of key genes composing the prediction model and clinical characteristics between the high-risk and low-risk groups. (E) Differences in immune processes among different risk groups. The vertical coordinates represent the relative scores of the relevant immune processes. (F) The differences in the relative abundance of various cell types in the lung tissues of the high-risk group and the low-risk group.


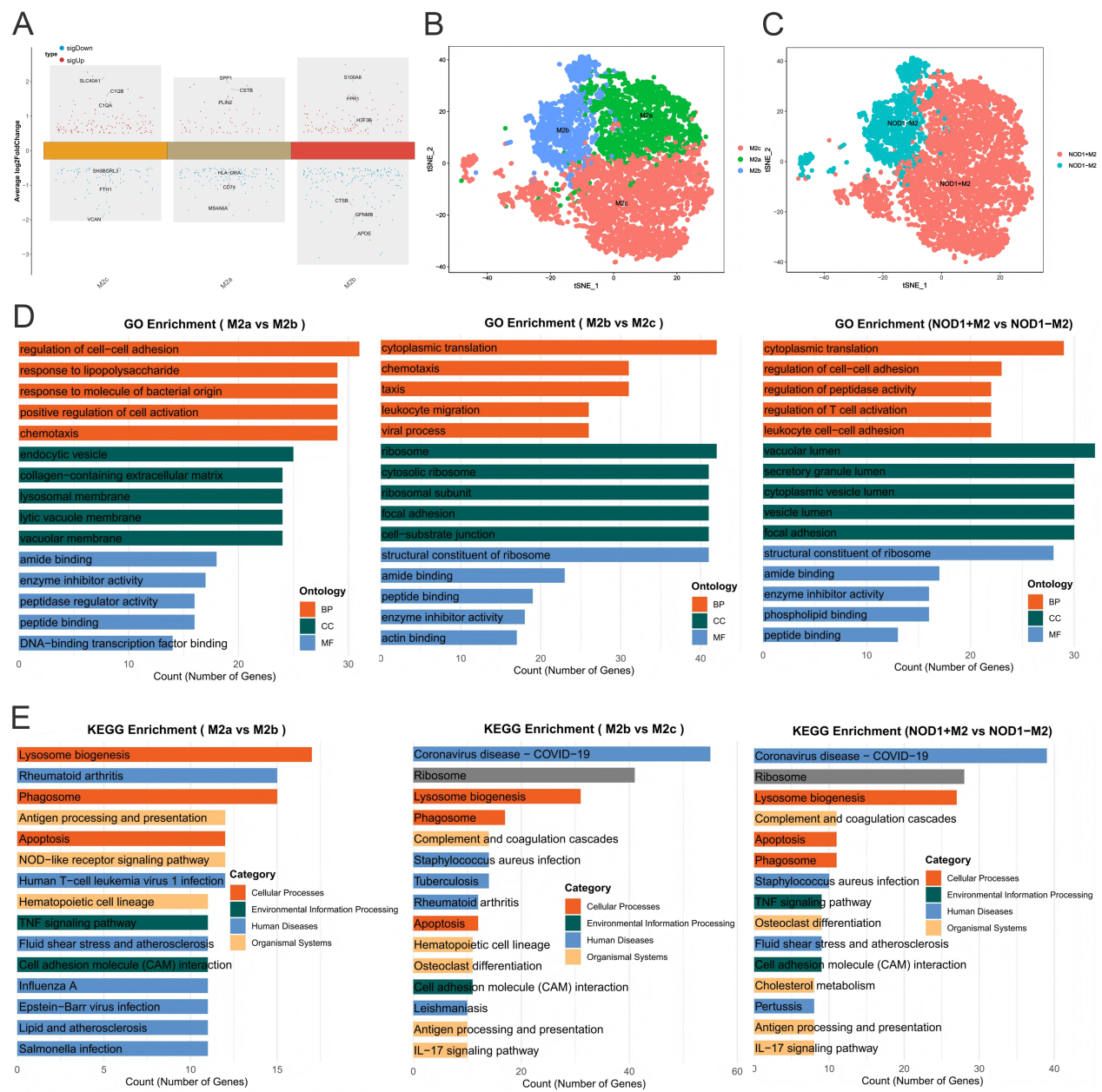


**Fig. S3 Macrophage subpopulation clustering and functional characterization.** (A) M2a、M2b、M2c macrophage subpopulations were identified based on distinct gene expression profiles and functional markers. (B) UMAP plot showing that macrophages can be classified into three subpopulations: M2a, M2b, and M2c. (C) The UMAP plot shows the distribution of NOD1⁻ M2 macrophages and NOD1⁺ M2 macrophages. (D) GO enrichment analysis results of differentially expressed genes among M2 macrophage subpopulations classified by different clustering approaches. (E) KEGG enrichment analysis of differentially expressed genes among M2 macrophage subclusters.


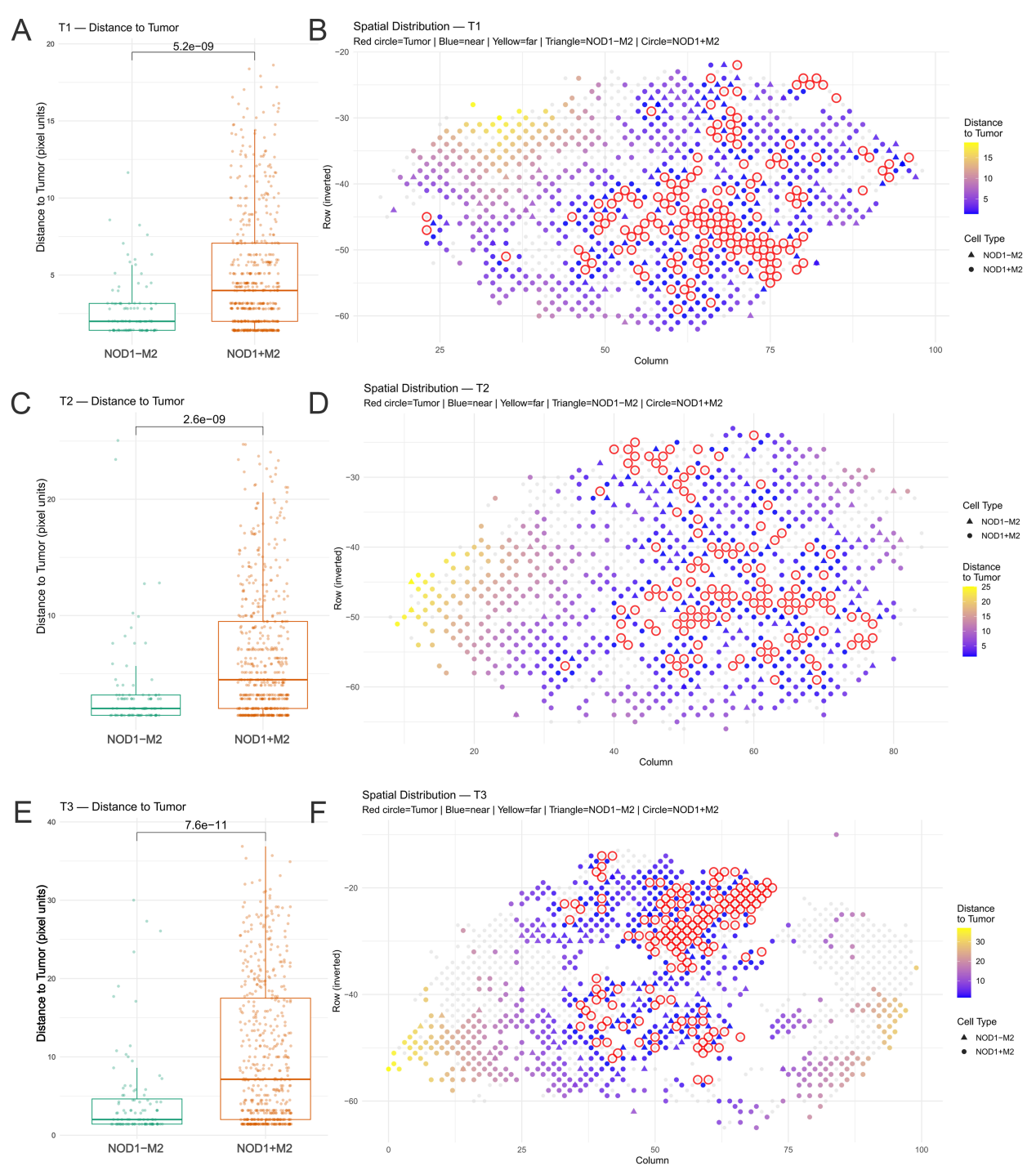


**Fig.S4 Spatial proximity analysis of NOD1⁺ and NOD1⁻ M2 macrophages to tumor cells in LUSC tissues.** (A) Box plot with individual data points showing the distance (in pixel units) from NOD1⁻ M2 macrophages and NOD1⁺ M2 macrophages to the nearest tumor cells in spatial transcriptomics sample T1. (B) Spatial distribution map of the corresponding sample T1. Triangles represent NOD1⁻ M2 macrophages, circles represent NOD1⁺ M2 macrophages, and red hollow circles denote tumor regions. Color gradient (blue to yellow) indicates the distance to the nearest tumor cell, with blue representing close proximity and yellow indicating greater distance. (C, E) Box plots for samples T2 and T3, respectively. (D, F) Spatial distribution maps for samples T2 and T3, respectively.


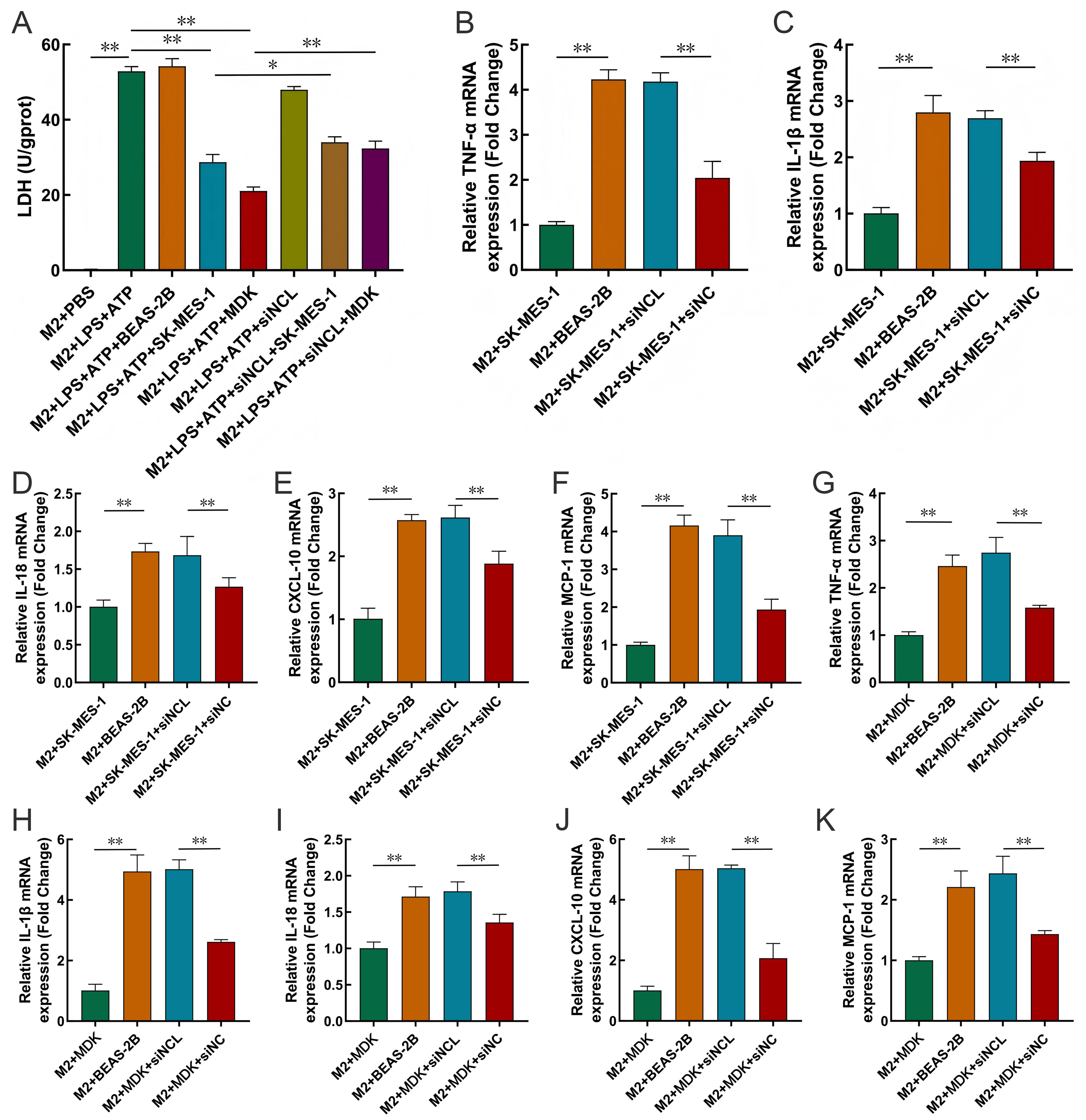


**Fig. S5 Effects of Cancer Cells and MDK on the Expression of Pyroptosis-Related Inflammatory Mediators in M2 Macrophages.** (A) The activity of LDH in each group of cell culture medium. (B) TNF-α mRNA expression levels under different treatment conditions. (C) IL-1β mRNA expression levels under different treatment conditions. (D) IL-18 mRNA expression levels under different treatment conditions. (E) CXCL-10 mRNA expression levels under different treatment conditions. (F) MCP-1 mRNA expression levels under different treatment conditions. (G) TNF-α mRNA expression levels under different treatment conditions. (H) IL-1β mRNA expression levels under different treatment conditions. (I) IL-18 mRNA expression levels under different treatment conditions. (J) CXCL-10 mRNA expression levels under different treatment conditions. (K) MCP-1 mRNA expression levels under different treatment conditions. * P < 0.05, ** P < 0.01.
